# Investigation of Surface Integrity Induced by Various Finishing Processes of AISI 52100 Bearing Rings

**DOI:** 10.3390/ma15103710

**Published:** 2022-05-22

**Authors:** Nabil Jouini, Philippe Revel, Guillaume Thoquenne

**Affiliations:** 1 Mechanical Engineering Department, College of Engineering, Prince Sattam Bin Abdulaziz University, Alkharj 16273, Saudi Arabia; 2 Laboratoire de Mécanique, Matériaux et Procédés (LR99ES05), École Nationale Supérieure d’Ingénieurs de Tunis, Université de Tunis, Tunis 1008, Tunisia; 3 Laboratoire Roberval de Mécanique FRE UTC-CNRS 2012, Université de Technologie de Compiègne, 60203 Compiegne, France; philippe.revel@utc.fr; 4 Centre Technique des Industries Mécaniques (CETIM), 52 Avenue Félix Louat, 60300 Senlis, France; Guillaume.Thoquenne@cetim.fr

**Keywords:** finishing processes, surface integrity, bearing steel, residual stresses, rolling contact fatigue

## Abstract

Surface integrity induced by finishing processes significantly affects the functional performance of machined components. In this work, three kinds of finishing processes, i.e., precision hard turning, conventional grinding, and sequential grinding and honing, were used for the finish machining of AISI 52100 bearing steel rings. The surface integrity induced by these finishing processes was studied via SEM investigations and residual stress measurements. To investigate rolling contact fatigue performance, contact fatigue tests were performed on a twin-disc testing machine. As the main results, the SEM observations show that precision hard turning and grinding introduce microstructural alterations. Indeed, in precision hard turning, a fine white layer (<1 μm) is observed on the top surface, followed by a thermally affected zone in the subsurface, and in grinding only, a white layer with 5 μm thickness is observed. However, no microstructural changes are found after sequential grinding and honing processes. White layers induced by precision hard turning and grinding possess compressive residual stresses. Grinding and sequential grinding and honing processes generate similar residual stress distributions, which are maximum and compressive at the machined surface and tensile at the subsurface depth of 15 μm. Precision hard turning generates a “hook”-shaped residual stress profile with maximum compressive value at the subsurface depth and thus contributes as a prenominal factor to the obtainment of the longest fatigue life with respect to other finishing processes. Due to the high quality of surface roughness (Ra = 0.05 μm), honing post grinding improves the fatigue life of bearing rings by 2.6 times in comparison with grinding. Subsurface compressive residual stresses, as well as low surface roughness, are key parameters for extending bearing fatigue life.

## 1. Introduction

In the field of bearing manufacturing, the finishing process chain of the bearing ring consists of grinding and raceway honing. Honing is applied post grinding to achieve the necessary surface characteristics. Hard turning is emerging as a competitive finishing process, offering potential benefits over the grinding process such as cost-effectiveness, manufacture of complex shapes, dry machining, and high flexibility [1,2]. The surface integrity induced by finishing processes significantly affects the functional performance (e.g., friction, corrosion, and fatigue) of machined components [3,4]. In order to improve the fatigue life of bearing rings, innovative hard machining processes have been developed. Hashimoto et al. [5] provide guidelines for the selection of fine finishing processes in order to obtain the desired performance of functions such as fatigue life. Maiß et al. [6] used a new hybrid process of hard turn-rolling to manufacture a bearing inner ring. As a result, the bearing fatigue life could be increased by a factor of 2.5. Revel et al. [7] proposed precision hard turning for the finishing of bearing steel components to improve the rolling fatigue life of bearing steel components. Arsalani et al. [8] used sequential hard turning, grinding, and ball-burnishing operations. They showed an improvement in the endurance limit with burnished pre-turned and burnished pre-ground samples compared to turned samples.

Rolling contact fatigue (RCF) has been commonly known as a key factor limiting bearing life [9]. Subsurface-originated spalling is recognized as one of the dominant mechanisms of failure in rolling element bearings [10]. Subsurface cracks are more likely to be initiated in the region of maximum shear stress under rolling contact below the surface and propagate toward the surface to form a surface spall [10,11].

Surface integrity is of particular importance when machining high-performance metals used for high-performance components in industries such as aerospace, biomedical, and automotive [3,12]. Surface integrity is characterized in terms of surface topography, as well as mechanical and metallurgical states of surface and subsurface layers.

Both hard turning and grinding processes introduce microstructural alterations in the surface layer known as white and dark layers [13,14,15]. These layers are visible under optical microscopy after being polished and etched or featureless under scanning electron microscopy (SEM). The white layer microstructure consists of nanocrystalline grains with grain size smaller than 100 nm [16,17] and is harder than the bulk material. The dark layer, which is located beneath the white layer, is generally softer than the bulk material. Two mechanisms, i.e., thermal and mechanical effects, are considered to be the major causes of white layer formation. Hosseini and Klement [18] investigated the characteristics and the formation of white and dark layers induced by hard turning. They showed that two different types of white layers exist: those that are either predominantly thermally or mechanically induced. Zhang et al. [19] showed that the white layer was formed by rapid austenite transformation and quenching process, and the dark layer was formed by the tempering process. Mao et al. [15] investigated the formation mechanisms and properties of the affected layer formed in grinding. They found that white layers may be generated when the grinding temperature is below the nominal phase transformation temperature. There are controversial opinions in the literature regarding how the white layer may impact the fatigue life of bearing steels. Guo et al. [20] showed that the machining-induced white layer can reduce the fatigue life of AISI 52100 steel by as much as 8 times. Opposite conclusions were achieved by Smith et al. [21], who carried out fatigue tests on AISI 52100 hardened bearing steel and concluded that there was no conclusive evidence suggesting that hard turning with a continuous white layer had a negative impact on axial fatigue performance.

Residual stresses can be either beneficial or detrimental for the components subjected to rolling contact fatigue life, depending upon their nature (compressive or tensile) and distribution at the subsurface depth. Shen et al. [22] used a three-ball-on-rod RCF test to experimentally assess the effects of retained austenite and residual stresses on the RCF of carburized AISI 8620 steel. Their results showed that the presence of compressive residual stresses is beneficial and increases RCF life. Matsumoto et al. [23] compared the fatigue life of ground and hard-turned bearing assembly. They found that the depth of compressive residual stresses is the major difference between hard-turned and ground surfaces. Indeed, hard turning produces compressive residual stresses in a deep subsurface, which improves the fatigue life of rolling bearings. Wang et al. [24] investigated the grinding mechanism of a bearing ring raceway and performed integrated modeling to control stress grinding. They showed that grinding can produce compressive residual stresses in the surface layer of the bearing ring raceway. Pape et al. [25] investigated the effect of residual stresses induced through production processes. They showed that the bearing fatigue life is improved due to the induced residual stresses on the bearing’s inner ring by sequential hard turning and deep rolling processes.

**Figure 1:**
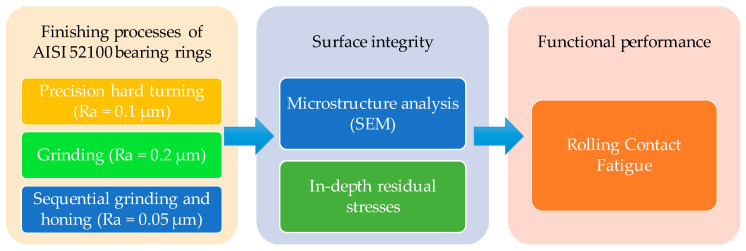
Workflow of the present study.

Rolling contact fatigue has been commonly known as a key factor limiting bearing life. Thus, it is necessary to improve the fatigue life of bearings by controlling the surface integrity of bearing raceways. In this respect, this paper investigates the surface integrity of AISI 52100 bearing rings finished by three kinds of finishing processes, i.e., precision hard turning, conventional grinding, and sequential grinding and honing, as well as the functional performance of the finished bearing rings. As shown in Figure 1, the surface integrity induced by these finishing processes was investigated via scanning electron microscopy investigations and in-depth residual stress measurements. Furthermore, contact fatigue tests were carried out on a twin-disc testing machine under two loading levels.

## 2. Materials and Methods

In this experimental work, the workpiece material was an AISI 52100 bearing steel (EN 100Cr6) ring. This steel material is widely used for the manufacture of bearing rings. The rings were heat-treated in order to obtain the desired hardness of 61 ± 1 HRC. AISI 52100 bearing steel material has a phase composition consisting of martensitic structure (95.3%), carbides (4.6%), and neglected retained austenite (<1%). The material properties of this steel are given in Table 1.

Surface integrity induced by three kinds of finishing processes was studied via SEM investigations and residual stress measurements. Hard turning tests were conducted on a high-precision machine, a prototype lathe positioned in an air-conditioned room, and developed for machining polish-mirror surfaces and hard steel [7,26]. The cubic boron nitrite (cBN) inserts and the machining process parameters used for the hard turning experiments have been precisely detailed elsewhere [7]. As the preconized surface roughness target from the bearing manufacturer, the bearing rings were finished by the Mcrorectif company, Saint-Étienne, France to a preferable target roughness average Ra of approximately 0.2 µm for grinding and 0.05 μm for sequential grinding and honing. The process parameters and roughness are controlled parameters. The roughness average (Ra) defined by ISO 4287 was measured using a scanning white-light interferometer Zygo^TM^ NewView 200 (Zygo Corporation, Middlefield, CT, USA). As can be seen, by applying honing after grinding, the surface roughness becomes smooth. The surface roughness of the precision hard-turned specimen was investigated in previous studies [27,28]. Indeed, a very low surface roughness value Ra = 0.1 μm is obtained in precision hard turning. Sequential grinding and honing processes allow us to achieve very low surface roughness Ra ≈ 0.05 with respect to precision hard turning.

**Figure 2:**
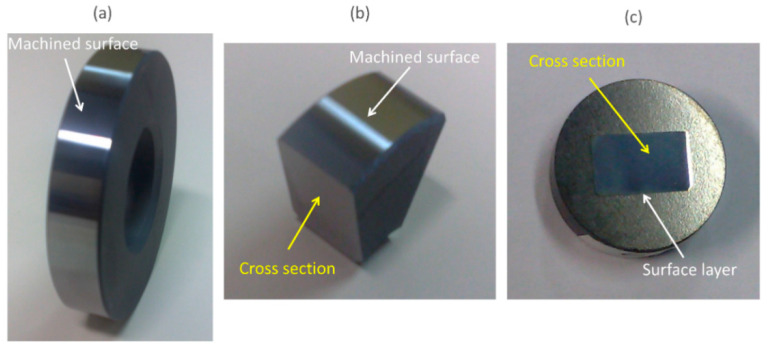
(**a**) Bearing ring finished by precision hard turning; (**b**) sectioned specimen; (**c**) mounted, polished, and etched specimen.

The specimens were prepared following standard metallographic techniques, as shown in Figure 2. Preliminary specimens were cut using an abrasive cutter and the Struers Discotom 5 machine (Struers, Ballerup, Denmark). The sectioned specimens were then hot mounted in Struers PolyFast conductive resin. Mounted specimens were then ground using 180-grit paper, followed by 320-, 600-, 800-, and 1200-grit papers in a Struers TegraPol-31 system and polished with diamonds. The polished surfaces were cleaned using an ultrasonic bath and then etched with 2% Nital solution for approximately 10 s. Microstructural investigations were carried out using the environmental scanning electron microscope XL30 ESEM-FEG (Philips Electron Optics, Eindhoven, The Netherlands) equipped with an energy-dispersive X-ray (EDX) analyzer.

**Figure 3:**
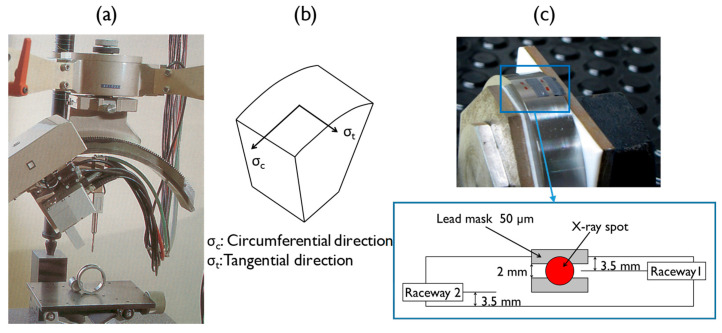
(**a**) Setup for the residual stress measurements; (**b**) residual stress measurement directions; (**c**) limiting device of X-ray beam with the raceway width.

The bearing rings were investigated regarding in-depth residual stresses. These residual stresses were measured by the X-ray diffraction method using a PRECIX robotic system (Figure 3a). The measurements were performed using Cr-Kα radiation diffracted at 2θ = 156° in the atomic plan {2 1 1} of the steel. Thus, these conditions give access to the strain localized at a depth of approximately 6 μm according to EN-15305 [29]. As shown in Figure 3b, the residual stresses were measured along the circumferential direction (σ_c_) and tangential direction (σ_t_). Both machined surface and in-depth residual stresses were measured. In the case of in-depth residual stresses, successive layers of material were removed by using chemical etching and electropolishing to avoid the reintroduction of residual stress. To evaluate residual stresses during the RCF test, the measurements were carried out on the bearing raceway, as shown in Figure 3c, thanks to a lead mask, which adapts the spot size of the X-ray beam with the raceway width.

**Figure 4:**
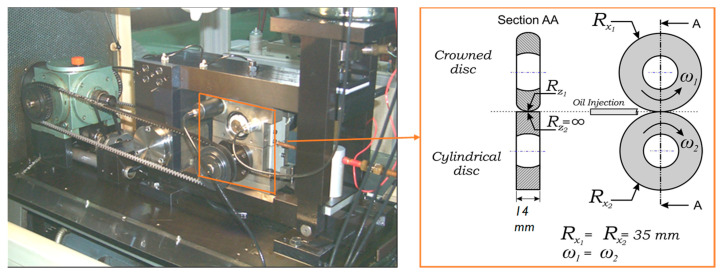
Twin-disc test machine and schematic representation of the geometry of ring specimens.

**Figure 5:**
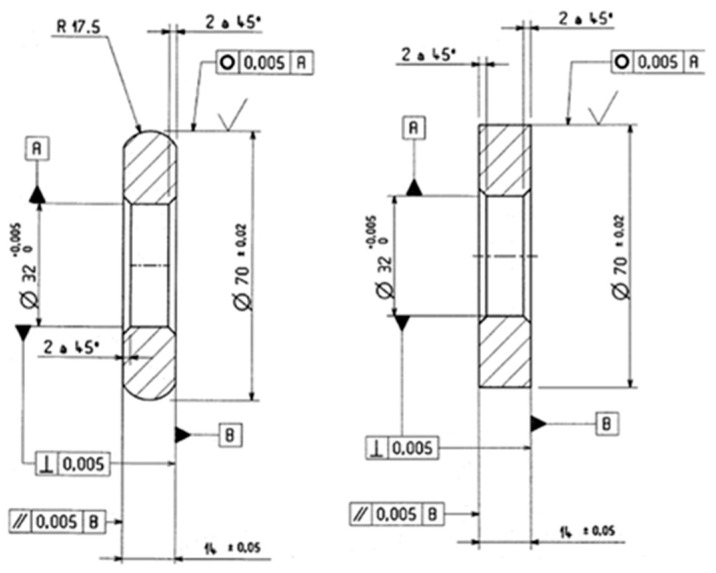
Drawing of ring specimens.

The rolling contact fatigue tests were performed on a twin-disc testing machine (Figure 4) available at CETIM Senlis, France. The contact is made between two discs, one being cylindrical and the other one being crowned with a crown radius of 17.5 mm, as shown in Figure 5. The two contacting discs have the same external radius of 35 mm and width of 14 mm. Tests were carried out under pure rolling conditions (slide-to-roll ratio = 0%), entrainment speed of 11 m/s, and lubricated with MobilGear 629oil injection. The applied normal load is 1100 daN, which is equivalent to the Hertzian contact pressure of 4.5 GPa. For each finished ring, two tests were performed under the same conditions. The twin-disc testing machine is equipped with sensors at the proximity of each disc to detect spalling occurrence. Thus, the test is stopped until spalling forms or when reaching 10 million cycles.

## 3. Results and Discussion

### 3.1. Microstructure Analysis

**Figure 6:**
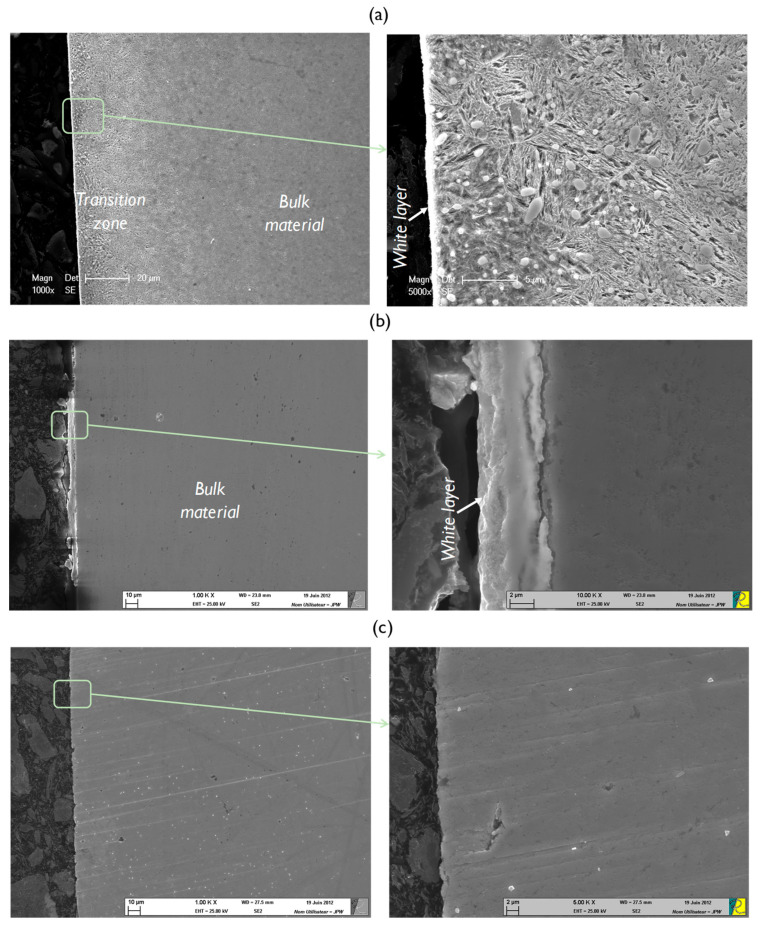
Micrographs of AISI 52100 steel after (**a**) precision hard turning, (**b**) grinding, and (**c**) sequential grinding and honing.

In order to highlight the metallurgical transformations, micrographic analyses were carried out on the cross-sectional samples of the three finishing processes under investigation. SEM observations show that precision hard turning and grinding introduce microstructural changes in the subsurface (Figure 6). Indeed, as shown in Figure 6a, after precision hard turning, a very thin white layer (<1 μm thickness) appears on the top surface, followed by a transition zone in the subsurface and then the bulk material. This very thin white layer can be attributed to the use of a new cutting tool for each machining test. Indeed, tool wear noticeably affects the white and dark layer thicknesses; a worn tool would generate a deep white layer [30,31]. This very thin white layer can also be beneficial for rolling contact fatigue performance. Schwach and Guo [32] showed that the white layer induced by hard turning is very detrimental to RCF life. Indeed, a component free of a white layer can have a life six times that of a white layer component. The transition zone was examined using energy-dispersive spectroscopy (EDS) to investigate the chemical composition and to determine the distribution of elements. The precipitated carbides are distributed spherical (Fe,Cr)_3_C carbides covering the transition zone. These carbides are observed in the white layer and the transition zone, but their number and size are not the same in different regions. Indeed, the carbides spread in the transition zone are more numerous. After grinding, as shown in Figure 6b, only a white layer with 5 μm thickness is observed above the bulk material, which is greater than that found after precision hard turning. Guo and Sahni [33] found that the thickness of the white layer induced by grinding is greater than those obtained by hard turning. In their micrographic analyzes, Barbacki et al. [34] showed that the white layer thickness varies from 0 and 2 μm, and the dark layer thickness varies from 0 to 5 μm in 17 samples machined by grinding. The absence of the dark layer under the white layer supports that the white layer formation is mainly due to mechanical work. Hosseini et al. [35] reported that the predominantly mechanically formed white layer is accompanied by compressive residual stresses. After sequential grinding and honing processes, as shown in Figure 6c, no microstructural changes are observed. Indeed, the honing process removes the white layer induced by the grinding process.

### 3.2. Residual Stresses

**Figure 7:**
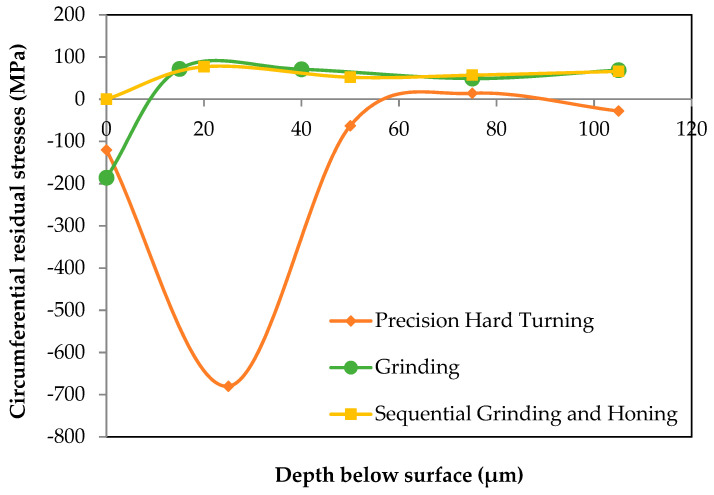
Residual stresses in circumferential direction.

**Figure 8:**
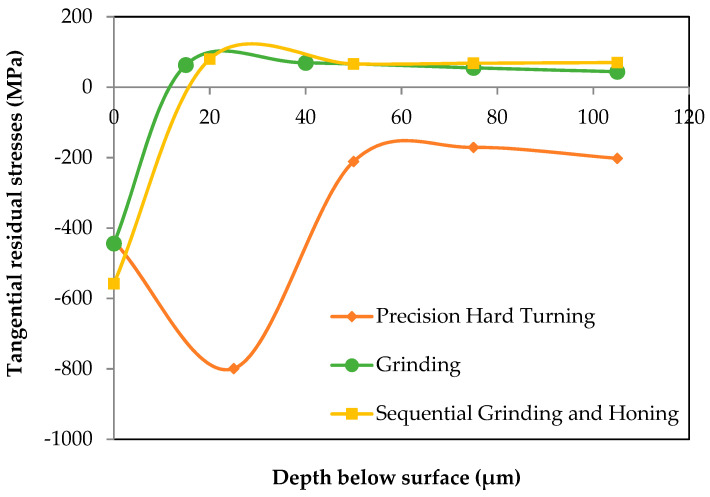
Residual stresses in tangential direction.

Figure 7 and Figure 8 illustrate the in-depth residual stresses in the circumferential and tangential directions induced by each finishing process. In the circumferential direction, residual stresses induced by grinding and by sequential grinding and honing have similar trends; they start out compressive at the machined surface (−186 and −290 MPa, respectively) and become tensile at the subsurface depth. Then, they are stabilized at around 50 MPa. The affected depth of residual stress is around approximately 15 μm. Mao et al. [15] showed that phase transformation in the white layer formed by grinding plays an essential role in the build-up of tensile residual stresses. This implies that the predominant factor is deformation, which leads to compressive stresses. As shown in the investigations of Pape et al. [36], residual stresses for ground and honed bearings are compressively closely adjacent to the surface (−500 MPa) and eliminated at a depth of about 20 μm. However, residual stresses induced by precision hard turning show significant differences from those induced by grinding and by sequential grinding and honing. Indeed, they exhibit a “hook”-shaped profile along the depth with maximum compressive value (−680 MPa) at the subsurface depth of 25 μm. Additionally, the affected depth of residual stress is greater for precision hard turning (around 50 μm). These results are in agreement with those reported by Smith et al. [21], who revealed similar differences in the stress states generated by hard turning and grinding processes. They found that the overall residual stress state is significantly more compressive on the hard-turned surface than on the ground surface. Matsumoto et al. [23] reported that the depth of compressive residual stresses is the major difference between hard-turned and ground surfaces. Considering the difference between the plastic deformation that takes place on the ground surface and that on the hard-turned surface, it is reasonable to expect that the compressive residual stress generated by hard turning is deeper, which may improve the fatigue life of rolling bearings. Otherwise, the results show that the white layers induced in both hard turning and grinding processes possess compression residual stresses. Hosseini et al. [35] showed that compressive residual stresses and decreased retained austenite content were found in the plastically created white layer. Residual stresses in the tangential direction (Figure 8) display similar trends to those described in the circumferential direction. Both grinding and sequential grinding and honing induce compressive and maximum residual stresses (respectively, −186 and −290 MPa) at the machined surface and become tensile at the subsurface depth. Residual stresses induced by precision hard turning start out compressive (−437 MPa) at the machined surface and exhibit a maximum compressive value of −800 MPa at the subsurface depth of 30 μm before stabilizing at the level. The residual stresses in the tangential direction are more compressive than those in the circumferential direction.

### 3.3. Rolling Contact Fatigue (RCF) Performance

**Figure 9:**
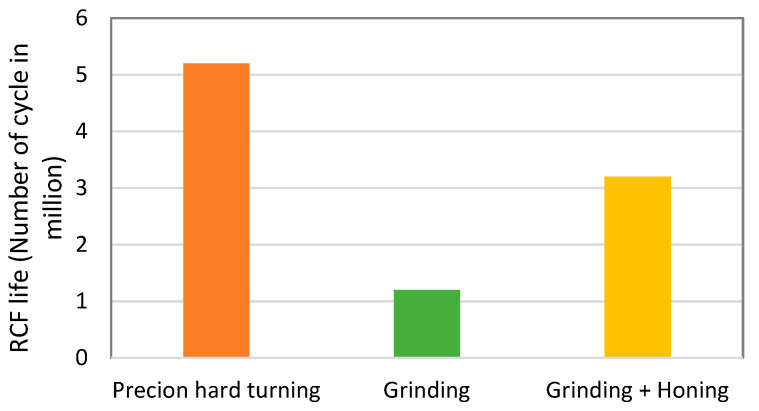
RCF life vs. finishing processes.

Surface integrity induced by finishing processes significantly affects the functional performance of machined components. To investigate rolling contact fatigue performance, contact fatigue tests were performed on a twin-disc testing machine. Figure 9 presents RCF life per million cycles as a function of the three finishing processes under investigation. For each finished ring, two tests were carried out on each specimen, i.e., each test on one raceway, under the same conditions. The rings finished by precision hard turning have the longest life (5.2 million cycles), while rings finished by grinding (Ra = 0.2 μm) have the shortest (1.2 million cycles). The rings finished by sequential grinding and honing (Ra = 0.05 μm) reach 3.2 million cycles. This shows that using the honing process after grinding improves the fatigue life of bearing rings by 2.6 times. Grinding and sequential grinding and honing have similar residual stress distributions, maximum and compressive at the machined surface and tensile at the subsurface depth. The enhancement of the lifetime is due to the high quality of surface roughness obtained by the grinding process. Precision hard turning offers better fatigue life due to the low surface roughness, as well as the residual stress state, which is compressive and maximum at the subsurface depth. The residual stress distributions are of considerable importance; several investigations have reported that compressive residual stresses induced by the manufacturing process can extend the fatigue life of bearings up to 2.5 times [20,23,37]. Low surface roughness and subsurface residual stresses are the key parameters for extending bearing fatigue life.

Furthermore, it is well known that residual stresses can be developed during the RCF test due to cyclic plastically [38]. Thus, changes in in-depth residual stresses during the RCF test of ring specimens finished by grinding were investigated due to their shortest fatigue life. The experimental simulation of rolling contact fatigue has two phases: a running-in phase called break-in and a bearing life phase. The running-in phase is estimated at 30,000 cycles under these test conditions [28]. After the running-in phase, the contact geometry is stabilized for the entire lifetime. Table 2 illustrates the Hertzian pressure for 600 and 1100 daN normal loads used in the experimental simulation and the RCF life of the ring finished by grinding. This table shows decreasing Hertzian pressure from 3.8 to 3.6 GPa for 600 daN and from 4.5 to 3.8 GPa for 110 daN. In addition, increasing the normal load decreases RCF life.

**Figure 10:**
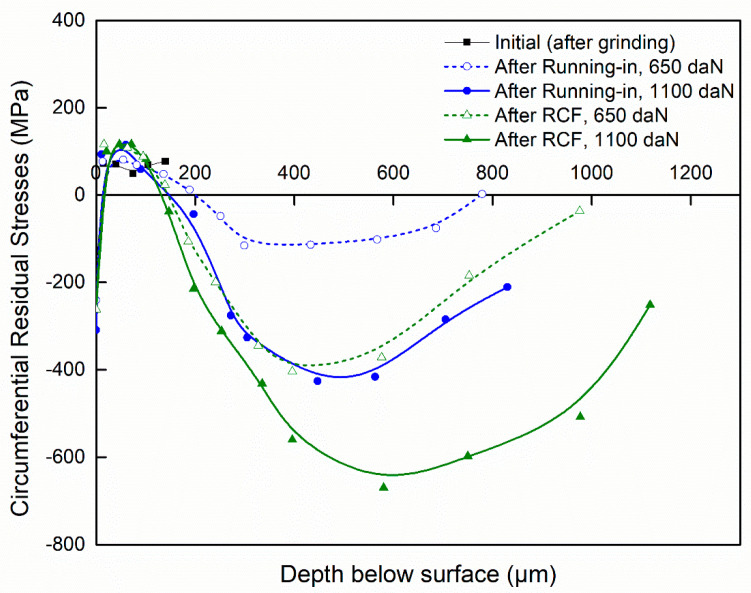
Circumferential residual stresses after different phases.

**Figure 11:**
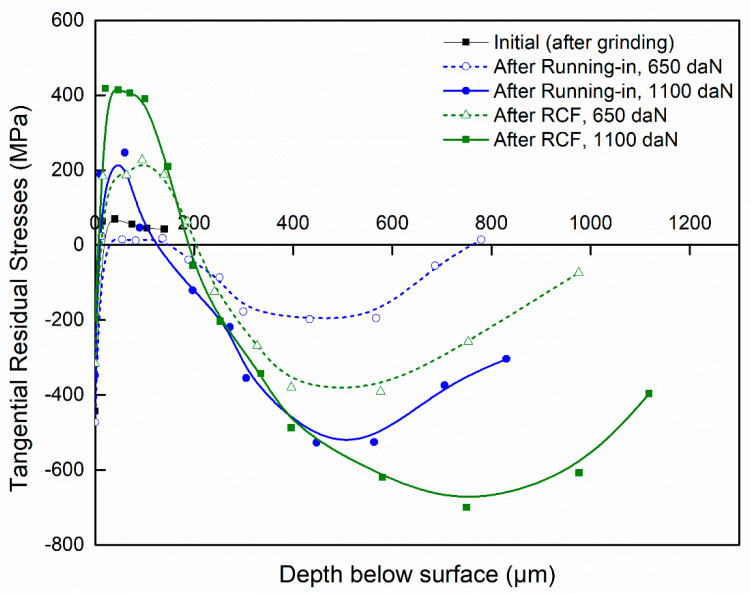
Tangential residual stresses after different phases.

Figure 10 and Figure 11 show the in-depth residual stresses with varying loads (650 and 1100 daN) after grinding (initial), after the running-in phase, and at the end of the RCF test. Before the RCF test, the ring specimen (initial) exhibits compressive residual stresses at the machined surface, originating from the machining operation, and tensile residual stresses at the subsurface depth. During the RCF test, the residual stresses changed from a moderate level of tensile residual stresses to compressive residual stress at the subsurface depth. Pape et al. [36] found a similar evolution, and the residual stresses remained constant after 10^6^ to 10^8^ revolutions.

Figure 10 shows that during the RCF test, the evolution of circumferential residual stresses in the first 140 μm subsurface depth is not noticeable. At a larger subsurface depth, the residual stresses changed from a moderate level of tensile residual stresses (around 50 MPa for initial) to peak compressive values. Indeed, after running-in with 650 and 1100 daN applied load, the maximum residual stresses are −116 MPa at 299 μm depth and −426 MPa at 427 μm depth, respectively. At the end of the RCF test, the maximum residual stresses are −116 MPa at 299 μm depth and −404 MPa at 396 μm depth. As shown in the investigations of Voskamp [39], the compressive residual stress peaks at a depth ranging from 0.1 to 0.5 mm as the cycles increase. This depth coincides with the maximum shear stress [11,39].

Figure 11 shows that at the shallow depth, ranging from 10 to 140 μm, the tangential residual stresses are tensile and become compressive at the subsurface depth with peak compressive values. Indeed, after running-in with 650 daN and 1100 daN normal load, the maximum residual stresses are −188 MPa at 433 μm depth and −527 MPa at 447 μm depth, respectively. After the end of the RCF test, the maximum residual stresses are −380 MPa at 396 μm depth and −700 MPa at 750 μm depth. As can be seen also that in both directions the peak compressive residual stress increases with increasing the normal load.

## 4. Conclusions

In this paper, the surface integrity of bearing rings induced by three finishing processes was investigated, and the following conclusions may be drawn:

- White layers induced by precision hard turning (<1 μm) and grinding (5 μm) possess compressive residual stresses.
- Subsurface compressive residual stress is the major difference between finishing processes. Grinding and sequential grinding and honing exhibit maximum and compressive residual stresses at the machined surface (−186 and −290 MPa, respectively) and tensile at the subsurface depth of 15 μm. Precision hard turning exhibits compressive residual stresses at the machined surface and maximum compressive value (−680 MPa) at the subsurface depth of 25 μm.
- Sequential grinding and honing improve the fatigue life of bearing rings by 2.6 times in comparison with grinding due to the improvement in surface roughness.
- Precision hard turning produces the longest fatigue life (5.2 million cycles) due to subsurface compressive residual stresses.
- The changes in subsurface residual stresses after the running-in process and after the spalling of bearing rings finished by grinding, which has the shortest fatigue life (1.2 million cycles), reveal that subsurface residual stresses are changed from a moderate level of tensile stresses to compressive ones, and such a change can positively affect the fatigue life.
- Subsurface compressive residual stresses originating from the finishing process, as well as low surface roughness, are the key parameters for extending bearing fatigue life.
- The future challenge will be the development of novel technology to enhance the fatigue performance of bearing rings. Thus, the effect of adding a honing operation after precision hard turning will be experimentally studied.

## Figures and Tables

**Table 1:** Material properties of AISI 52100.

Parameter	Value
Chemical composition (wt%)	1.040% C; 0.32% Si; 0.34% Mn; 0.04% Ni; 1.52% Cr; 0.007% Mo.
Young’s modulus E (GPa)	210
Poisson ratio	0.3
Hardness (HRC)	61 ± 1

**Table 2:** Hertizian pressure variations after running-in and RCF life of rings finished by grinding.

	Normal Load (daN)	
	600	1100
Initial Hertzian pressure (GPa)	3.8	4.5
Hertzian pressure after running-in (GPa)	3.6	3.8
RCF life (number of cycles)	2.6 × 10^6^	1.3 × 10^6^

## Data Availability

Data are available on reasonable request.
